# Patient Perceptions on the Advancement of Noninvasive Prenatal Testing for Sickle Cell Disease among Black Women in the United States

**DOI:** 10.1080/23294515.2024.2302996

**Published:** 2024-02-13

**Authors:** Shameka P. Thomas, Faith E. Fletcher, Rachele Willard, Tiara Monet Ranson, Vence L. Bonham

**Affiliations:** a School of Public Health, Harvard University, Boston, Massachusetts, USA; b National Institutes of Health-National Genome Research Institute, Bethesda, Maryland, USA; c Center for Medical Ethics and Health Policy, Baylor College of Medicine, Houston, Texas, USA; d School of Public Health, University of Washington, Washington, Seattle, USA

**Keywords:** Noninvasive prenatal testing, Black women, sickle cell disease, reproductive health equity

## Abstract

**Background:**

Noninvasive prenatal testing (NIPT) designed to screen for fetal genetic conditions, is increasingly being implemented as a part of routine prenatal care screening in the United States (US). However, these advances in reproductive genetic technology necessitate empirical research on the ethical and social implications of NIPT among populations underrepresented in genetic research, particularly Black women with sickle cell disease (SCD).

**Methods:**

Forty (*N* = 40) semi-structured interviews were conducted virtually with Black women in the US (19 participants with SCD; 21 participants without SCD) from June 2021 to January 2022. We employed a qualitative approach to examine the study participants’ perceptions of the potential advancement of NIPT for screening SCD in the fetus. Data were analyzed using NVivo 12 qualitative software.

**Results:**

The themes revealed the complexities involving the intersectional lived experiences of SCD, prenatal care, lack of synergy among health providers, and NIPT decision-making. The results further revealed that even when Black women have shared commonalities in their lived experiences while navigating SCD and motherhood, their perceptions of NIPT screening technologies are divergent.

**Conclusion:**

Expanding the ethical discourse on the social implications of NIPT is critical to fully elucidate how Black women perceive NIPT’s utility, particularly as NIPT advances to screen for SCD in the fetus. Neglecting to include Black women with genetic conditions in empirical studies on NIPT may contribute to ongoing health inequities and limit and constrain reproductive choices among Black women with and without SCD.

## Background

Noninvasive prenatal testing (NIPT) for genetic conditions is designed to use cell-free DNA (cf-DNA) in maternal plasma to screen for fetal genetic abnormalities during pregnancy (American College of Obstetricians and Gynecologists’ Committee on Practice Bulletins—Obstetrics, Committee on Genetics, and Society for Maternal-Fetal Medicine 2020). Currently, the American College of Obstetricians and Gynecologists (ACOG) recommends offering all pregnant people NIPT screening for aneuploidies (e.g., sex chromosomal characteristics and trisomy 21, 13, and 18, commonly known as Down syndrome), regardless of genetic risk or reproductive age. Noninvasive screening is cost-effective (Ohno et al. 2013) and reduces procedure-related pregnancy losses (Fairbrother et al. 2016), compared to invasive diagnostic procedures, such as amniocentesis or chorionic villus sampling. Considering the clinical benefits, NIPT is increasingly being implemented as part of routine prenatal care screening in the US. However, as a result of these advances in reproductive genetic technology, there is a need for more empirical research on the ethical and social implications of NIPT among populations underrepresented in scientific research, including Black women with genetic conditions, such as sickle cell disease (SCD) (Ravitsky et al. 2021; Thomas et al. 2023), which is the primary focus of this study.

Scientific advancements for NIPT utilization aims to permit safe in-utero screening for SCD and β-thalassemia disease in the fetus through cf-DNA (Erlich et al. 2022; van Campen et al. 2020; Westin et al. 2021, 2022). NIPT for SCD screening technology is in experimental stages and not yet approved by the US Food and Drug Administration (Welcome to Unity n.d., 4). However, it is commercially available in CLIA-approved laboratories (Clinical Improvements Amendments Act of 1988) and expected to be used in clinical settings across the US (UNITY Carrier Screen— Clinical Test—NIH Genetic Testing Registry—NCBI n.d.; Cutts et al. 2019; van Campen et al. 2019). Critical gaps, however, exist regarding how Black women with genetic conditions negotiate decision-making during prenatal screening, while also navigating the possible advances of NIPT for SCD in the fetus. Since Black women are disproportionately affected by SCD and are underrepresented in genetic research on NIPT (Thomas et al. 2023), this qualitative study examines Black women’s attitudes, perspectives, acceptability, and willingness to participate in NIPT screening. To our knowledge, this is the first empirical NIPT study to systematically engage Black women with and without SCD in the US context.

## Methods

### Study design

Forty (*N* = 40) semi-structured interviews were conducted virtually with Black women (19 with SCD; 21 without SCD) from June 2021 to January 2022. Table 1 presents the demographic characteristics of the participants. We purposefully included Black women across all reproductive statuses (e.g., no children by choice, pregnancy loss, infertility, or birth mother) to assess a broad range of perceptions of NIPT’s utility and its potential advancement in screening for fetal SCD. The inclusion criteria were women between the ages of 18 and 51 years, self-reported as Black, and with or without SCD. Pregnant women were excluded during data collection to maintain a hypothetical stance as the core part of our qualitative strategy. However, we created space for study participants to reflect on any prior pregnancies and prenatal experiences during the interview process. Each recruited individual underwent a screening survey to determine their eligibility to participate in the study. Each participant completed a demographic survey and a semi-structured interview which lasted between 60 and 90 min.

This qualitative study draws extensively from phenomenology, which focuses on the premise that patients are individuals, not cases or variables, who have an experiential world and lived experience (Charon 2006; Murphy et al. 2017). This approach to collecting women’s narratives requires researchers to allow patients to use their own language to describe clinical phenomena and gain a more complex understanding of their medical preferences and decisions in the context of their social lives. Conducting phenomenological interviews provides an opportunity to engage patients deeply rather than minimize their clinical encounters with a singular focus, such as the basic or routine mechanics of their illness experiences.

### Interview guide and clinical vignette

The interview guide presents a clinical vignette (see Online Appendix A), which was designed by our internal research team and reviewed by our external clinical consultants. These experts include a study nurse, genetics counselor, hematologist, and an obstetrics-gynecology (OBGYN) physician specializing in high-risk pregnancies and sickle cell disorders. The interview guide was developed based on themes identified in the literature review related to prenatal care, maternal health, social experiences, sickle cell disease, and genetic technology assessment. First, we sought to measure pre- and post-knowledge of the current use of NIPT among the study participants. We administered a baseline assessment of the participant’s current knowledge of NIPT and then exposed them to the ACOG NIPT patient education materials (2020) to assess their viewpoints (see Online Appendix B). The ACOG patient education materials used as a part of our interview protocol are based on the current use of NIPT to screen for trisomy 13, 18, and 21 (most commonly known as Down syndrome) in the US. The patient education materials were used (1) to expose our study participants to material that is currently being disseminated by a national organization; and (2) to elicit patient perceptions about NIPT-based health conditions.

The clinical vignette was designed as part of the interview guide (see Online Appendix A) to provide study participants with a hypothetical scenario to examine their perceptions of NIPT for fetal SCD screening. The purpose of the clinical vignette was to examine how the participants would advise a hypothetical patient on NIPT decision-making (Ulrich and Ratcliffe 2007). The clinical vignette involved a hypothetical story of a Black woman living with SCD who was seven weeks pregnant when receiving NIPT test results for her fetus, potentially having SCD. The study participant advised the hypothetical patient, allowing our research team to examine whether and how these views aligned with their own beliefs and perceptions. Additional decisions to consider after receiving NIPT results involved: (1) considering more invasive testing for definitive, invasive SCD diagnosis, which could increase the chances of pregnancy loss; (2) opting to keep NIPT’s predictive screening results without further invasive diagnostic testing; and/or (3) deciding on early termination of the fetus without further noninvasive screening or invasive testing. Although all pregnant women with NIPT results are offered these options, we are particularly interested in understanding how these options affect Black women with SCD.

One of the challenges of prenatal screening is the lack of a standardized interpretation of the results (Skotko et al. 2019). We exposed each participant to the ACOG NIPT patient education materials at baseline, which explains the concepts of “false positives” and “false negatives.” The clinical vignette was framed using language that patients conventionally hear when receiving results, as recommended by our external team of clinical consultants.

### Data analysis

Using an inductive analytical approach, data were analyzed using NVIVO 12 qualitative software to organize the responses from the interview transcripts (About NVivo, n.d.). The initial coding process was conducted by ST and RW, who coded separately using a codebook and then iteratively compared the codes and notes to merge into one master coding file. Our team of three researchers (ST, RW, and TR) met periodically to identify the patterns and themes in the data. The data were dichotomized into two broad groups: SCD and non-SCD, which included four subgroups: (1) SCD women with biological children, (2) SCD women without biological children, (3) non-SCD women with biological children, and (4) non-SCD women without biological children. Audio recordings and field notes were reviewed to cross-reference thematic points and capture additional nuances.

### Ethical approval, recruitment, and informed consent

The NIH Institutional Review Board (IRB) approved this study as exempt (IRB 000415). We recruited participants from SCD advocacy organizations. However, to capture a wider range of respondents, we relied substantively on respondent chain sampling to recruit more study participants who did not frequently engage with SCD advocacy organizations. Overall, it should be noted that this study was conducted virtually during the pandemic and consent was obtained verbally.

## Results

### Study participants

Forty Black women (*N* = 40) across the US participated in this study. Table 1 illustrates the sociodemographic characteristics of the study participants, highlighting the diversity of Black women at the national level. For the purposes of this analysis, we did not obtain the sickle trait carrier status of all the participants; thus, we did not report differences related to carrier status in this analysis. Our results capture patients’ perceptions of NIPT’s current ability to screen for conditions such as Down syndrome, and these perceptions were used to understand fetal screening for SCD.

**Figure 1.**
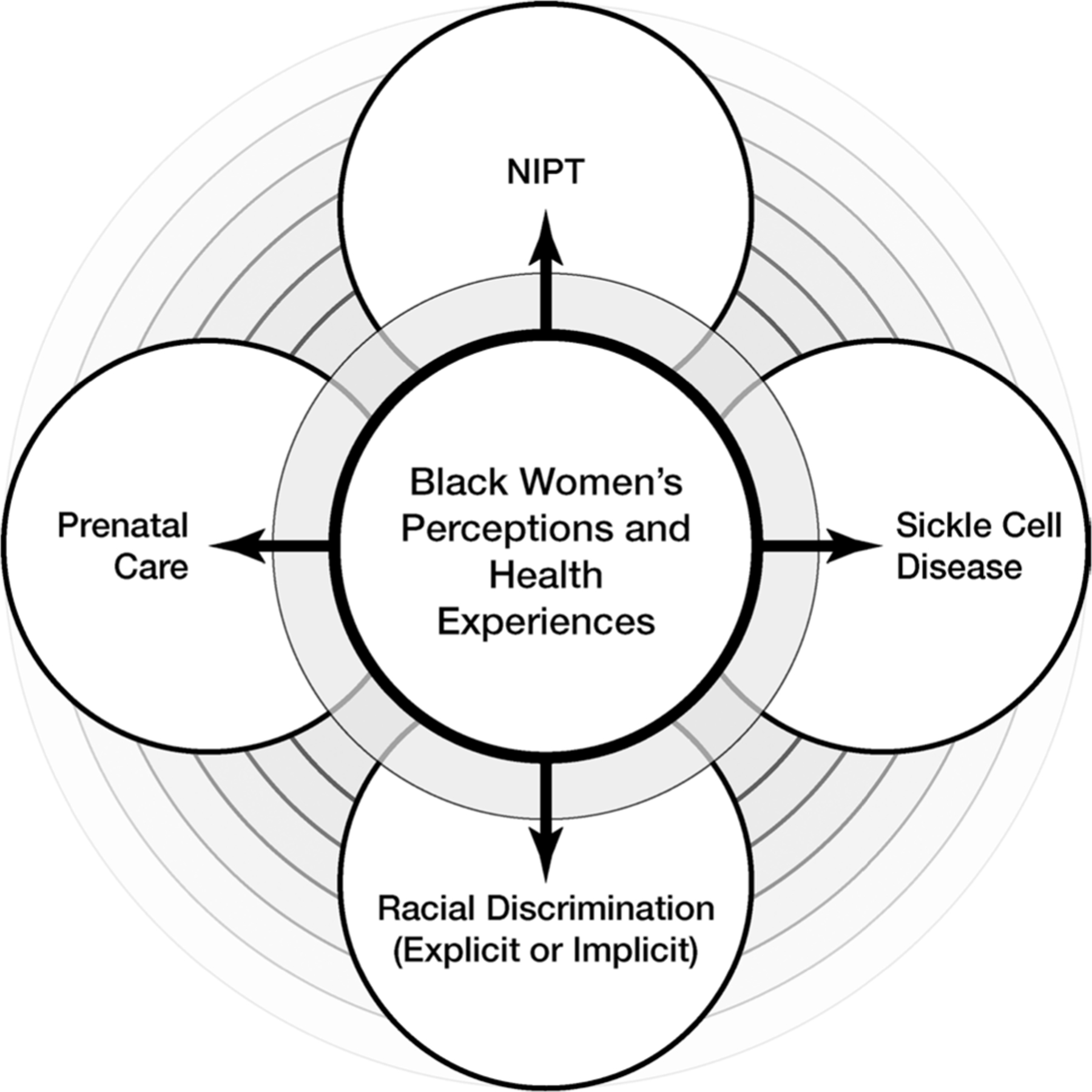
Conceptual model connecting NIPT, prenatal care, and SCD with the intersectional importance of examining Black women’s perceptions and health experiences.

Figure 1 illustrates our model conceptualizing the lived experiences of Black women with and without SCD who also navigate their reproductive healthcare across the US healthcare system (Roberts 1997). We argue that focusing on Black women’s reproductive health narratives is important for promoting reproductive equity in the context of advances in NIPT. Thus, we need to ethically consider how Black women with genetic conditions negotiate and navigate life-altering decisions related to predictive screening tests.

The results and quotes refer to the predictive value of NIPT and the advancement of NIPT’s potential to screen for SCD in utero. The results below include perspectives captured in the patient’s own words across three categories: (1) pre-NIPT educational material exposure, (2) post-NIPT educational material exposure, and (3) perceptions after the clinical vignette scenario.

### Category one: Pre-NIPT educational material exposure

#### NIPT’s main concern should be safety for the mother and fetus.

We examined how respondents described the meaning of NIPT and NIPT for fetal SCD at baseline. Some respondents had general knowledge about NIPT, particularly respondents who gave birth after 2011 (when NIPT became clinically available in the US) and mentioned having received NIPT during their first or second trimester during prenatal care. Regardless, we defined baseline knowledge as the respondent’s prior knowledge of NIPT at the beginning of the interview, without our research team prompting NIPT’s clinical definition and/or without offering exposure to NIPT educational material from our interview guide. At the beginning of the interview session, we asked, “What does noninvasive prenatal testing sound like and/or mean to you?”

Noninvasive means that it’s not going to impact the fetus or the child that one person is carrying. ~Respondent H: Mother, SCD-Group

That my child will be safe, that my fetus would be safe, you know, my baby would be okay. That’s always the main concern, especially as a mother—especially if it’s your first time being a mother—you want to know that you’re taking every precaution to bring your child into this world as healthy as possible, not have any—you know, any issues, any complications during your pregnancy. So, anything that will kind of lean towards that. ~Respondent B: Mother, Non-SCD Group

Something that’s outpatient….and by that, I mean, does not put the mother or child at risk. It causes minimal pain and after effects. Yeah, I think the flashing word for me is risk factors. So, for me, something that is minimally invasive has the least risk factors but it’s still effective. ~Respondent A No Children: Non-SCD Group

### Category two: Post-NIPT educational material exposure

#### Perceptions of NIPT results as predictive.

After exposing the respondents to the NIPT Educational Material (Online Appendix B) from our interview guide, they discussed their reactions to the predictive value of NIPT results. Most respondents were expressive about the predictive value of NIPT screening, particularly as it relates to the potential advancement of NIPT screening for fetal SCD. The respondents shared their perceptions of the predictive aspects of NIPT screening results, such as negotiating how predictive results would affect women currently living with SCD.

…this does not mean your child has this disease either way. This is a predictive value, like—yes, the risk is greater if this is positive, but that still doesn’t confirm that [SCD] is what your child has.

~Respondent J: Mother, SCD Group

Personally, just from what I’ve just heard about [NIPT], I personally don’t really like it. I would say that because it really doesn’t tell you anything. The positive or negative, either one is just predictive…I don’t like that the results are pretty much based on maybes and not definite positive or definite negative.

~Respondent E: Mother, SCD Group

#### NIPT predictive results are still useful for persons with and without genetic conditions.

A respondent from the non-SCD group, who grew up as a witness and caretaker of family members with SCD, discussed the usefulness of such predictions. Although this respondent acknowledged the limitations of the NIPT predictive results, she nevertheless opted for more invasive testing for a more definitive diagnosis. The respondent described requesting amniocentesis during her previous pregnancy and relying on diagnostic reproductive genetic technologies.

[NIPT] would be a useful option before…more invasive testing, although, because it’s predictive, I…would have gone for…definitive testing, regardless of what [NIPT] results were. ~Respondent C, Mother with SCT, Non-SCD Group

#### NIPT predictive results are still a stressful option for persons with and without genetic conditions.

Another respondent from the non-SCD group, who had a child with SCD, had an opposing perspective on the utility of the NIPT. She did not perceive the screening results as “useful,” but perceived them as stressful. This particular respondent also shared that her lived experiences with fertility and pregnancy were already challenging, and thus she was curious if receiving such screening results would further prevent her from choosing to enjoy her pregnancy. This respondent also confirmed that she eventually gave birth to her first child (without SCD) and later to a second child (with SCD).

The options are stressful. I wouldn’t want to do it. I want to be able to just enjoy my pregnancy. And I definitely would not want further testing. Respondent J, Non-SCD Group

#### Category three: Clinical vignette reactions and responses.

Our clinical vignette captures the story of a hypothetical patient, a Black woman with SCD, who was seven weeks pregnant when she received “false-positive” NIPT results for SCD in the fetus. In alignment with the ACOG NIPT Education Material (Online Appendix B), we based the hypothetical patient’s optional choices on the following: (1) considering more invasive testing for a definitive, invasive SCD diagnosis, which could increase the chances of pregnancy loss; (2) opting to maintain the NIPT’s predictive results without further invasive diagnostic testing; and/or (3) deciding for early termination of the fetus without further noninvasive screening or invasive testing.

Our clinical vignette allowed us to provide all respondents (both SCD and non-SCD groups) with an opportunity to react and respond to the NIPT’s potential to screen for fetal SCD in a hypothetical scenario. A respondent perceived the material as limiting, noting that the hypothetical patient had limited choices. She recommended that the hypothetical patient should still assume that the fetus will have sickle cell disease without putting herself at risk for further invasive testing.

#### Negotiating the predictive assumption that the fetus is going to have sickle cell disease.

[the hypothetical patient] doesn’t really have too great of options here…I would just say continue on in the pregnancy without the invasive procedure with the assumption that the baby is going to have sickle cell.

~Respondent F: Mother, Non-SCD group

After clinical vignette exposure, another respondent reflected on her lived experiences regarding the restriction of birthing choices because of an SCD diagnosis. This is an important concern for Black women, who already face limited and poor-quality reproductive healthcare options in US hospitals. The respondent was also unclear as to whether the screening results for SCD, even in a hypothetical situation, would further restrain her birthing choices because she was considered high-risk.

I wanted a midwife…but they wouldn’t accept me. The midwives—the nursing centers I talked to; they didn’t accept me because it was high risk. And so, they—I guess, they didn’t want that liability. And I didn’t like the fact that I was automatically categorized as high risk just because I had a diagnosis of sickle cell. it shouldn’t just be a blanket diagnosis.

~Respondent K: Mother, SCD Group

#### Issues with NIPT’s current framing of sickle cell disease as an “abnormality.”

One respondent from the SCD group (see below) described how it did not matter whether her child had SCD. This is not to say that the respondent was apathetic about her child having a genetic condition. Rather, the respondent did not subscribe to the framing of SCD as an “abnormality,” particularly given the notion that she also has SCD and still describes herself as living a functional and capable life. This respondent applied the clinical scenario to her own lived experiences with SCD.

So, like, when I was pregnant with my oldest son, I was offered amniocentesis—you know, and I didn’t want to do it just because I knew it was a risk factor to it, and I didn’t care one way or the other if he had sickle cell. I knew it was a risk that I was taking when I got pregnant, and I just didn’t want the test.

~Respondent H: Mother, SCD Group

#### Shared lived experiences, yet divergent perceptions of NIPT utility.

The majority of respondents who were mothers from the SCD group may have commonalities in their lived experiences. Despite these commonalities, respondents had divergent perceptions of the utility of NIPT screening for SCD. In other words, our results revealed that despite sharing common lived experiences such as being Black women, mothers, and/or having SCD, it cannot be assumed that individuals with these similarities hold similar views on reproductive genetic technology. This is a very important result in our study because it not only disentangles the conventional homogenization of Black women in empirical NIPT studies but also captures the breadth and depth of patient perceptions that are often underrepresented.

Below, we highlight the contrasting viewpoints on prenatal genetic technology among women who share similar life experiences.

#### In favor of NIPT screening for SCD

I would want to know definitely…because [SCD] is not something you want to see someone go through—you do not want to see your child go through.

~Respondent V: Mother, SCD Group

#### Opposed to NIPT screening for SCD

what’s the point? I mean, what are they trying to achieve; it’s going to affect the parents more than anybody, so I just am curious to know what is there to gain by…the medical world having this information.

~Respondent Z: Mother, SCD Group

Despite wide-ranging views on NIPT, respondents (from both the SCD and non-SCD groups) expressed heightened challenges around clinical decision making for Black women due to historical and ongoing adverse experiences in the US healthcare system. These realities persist irrespective of NIPT advancements for SCD.

Respondents from both groups expressed their awareness of reproductive health inequities beyond the clinical use of the NIPT.

I think this is a topic that deserves a great deal more spotlight than it gets and is emerging as problematic but has historically and contemporarily been problematic for a very long time. I don’t think that you will meet any Black woman who [didn’t] either have their own experience or have heard of an experience from another Black woman in her circle that doesn’t include some really difficult stories of mistreatment, malpractice, or medical negligence. ~Respondent A: No Children, Non-SCD Group

I have a strike against me as a Black person. Another strike as a woman…and then sickle cell. The burden and mistreatment during labor and delivery.

~Respondent Z: Mother, SCD Group

I’ve heard testimonies of other…African American women, who did not have sickle cell who experienced those [same] types of biases… I did actually do a consultation with the other doctor that my O.B. had partnered with and he allegedly had sickle cell knowledge. But when I sat with him to do the consultation, it was about five minutes long. It was literally five minutes long. And I asked him, I said, “So, what do you think I should do differently than a traditional person who’s pregnant?” And he didn’t really have much to share. The only difference was that I came to the doctor more often…there was no different treatment…it was just more monitoring…and jump through these extra hoops. ~Respondent K: Mother, SCD Group

## Discussion

### Need for increased empirical research to assess Black women’s barriers and concerns around NIPT

Black women have not been prioritized in the empirical NIPT research (Thomas et al. 2023). In the larger context, SCD is a major life-threatening genetic condition (Power-Hays and McGann 2020), in which pregnancy has been associated with exacerbating SCD pain crises (Smith-Whitley 2019). SCD disproportionately affects Black populations, and Black women with SCD are six to ten times more likely to die from pregnancy-related complications. Those women who become pregnant are inevitably placed in high-risk prenatal categories (Boakye et al. 2021; Elenga et al. 2016), which supports our finding that reproductive choices are limited for women living with SCD. Indeed, NIPT’s potential expansion to screen for SCD in prenatal cfDNA has been reported to be “a promising new technology” that can screen for prenatal results, without requiring paternal screening or blood samples from the biological father (Westin et al. 2022), but more critical research is needed for what this means specifically for Black women’s mental health and reproductive choices. The development of NIPT technology to screen for SCD using cfDNA is expected to reduce the potential harm to the mother and fetus (Gerovassili et al. 2006; van Campen et al. 2020). In the US, invasive tests such as amniocentesis and chorionic villus sampling are the current prenatal tests used to definitively diagnose SCD, which also increase the risk of procedure-related pregnancy loss (Bianchi 2015; Gerovassili et al. 2006). Advancements in NIPT have revealed ethical and social implications that should be increasingly examined, particularly as NIPT utility is increasing. Our results illustrate that further research is needed to elucidate how minoritized patients with multiple identities assess NIPT utility and what decisions they should consider regarding the use of this prenatal screening method.

### Important to consider reproductive choices amid NIPT screening and its ethical complexities

One of the challenges, for example, with NIPT’s potential advancement in screening for SCD is whether the results will broaden or constrain birthing choices for women with genetic conditions. Based on our results, although fetal screening for SCD relies on blood samples using cell DNA, our study participants highlighted that these predictive screening measures can create additional stress and psychological harm for Black women who are already navigating high-risk pregnancies. Thus, decision-making processes can still be psychologically invasive, which is a serious ethical concern when reproductive choices are further constrained for Black women with genetic conditions. This is particularly important for Black women, who simultaneously navigate the lived experiences of race and racism while also navigating racialized genetic conditions such as SCD. In other words, as Black women in the US disproportionately face serious maternal morbidity and mortality from preventable circumstances (Adams and Thomas 2018; Thomas 2022), we must consider the ethical complexities surrounding regulating genetic technology for SCD. We also delineate, through normative and empirical bioethics methods, how reproductive decisions are made based on predictive results from screening tests in the context of a healthcare system that fails to meet the needs of populations constrained by structural inequities.

Reproductive health disparities have worsened, severely impacting the lived experiences of Black women in the US (Thomas 2022), and genetic technological advancements must meet the needs for fundamental health equity to emerge. This is evident through the additional complexities that Black women face regarding lack of access to quality healthcare services, racial discrimination, implicit and explicit bias in clinical encounters, and residential segregation (Bell et al. 2006; C. A. Collins and Williams 1999; ProPublica and Montagne 2017). Thus, people with lived experiences as minoritized individuals move through constrained contexts (either by society or illness). This means that options after the return of NIPT results are not just about informed consent or individual decision-making regarding the pregnancy. Rather, reproductive choices (made before or after genetic screening) are an extension of the social and political environment, which can either increase or decrease well-being, longevity, and quality of life (Cazenave 1979). We argue that neglecting to capture this nuance may result in a well-intended and noninvasive procedure being perceived by Black women with and without genetic conditions as socio-psychologically invasive.

For ethical considerations, NIPT advancements require deeper inquiries to advance reproductive equity, such as: *How might the lived experience of racial discrimination influence Black women’s perceptions of NIPT? How might Black women’s reproductive choices, including that of early termination be restricted in post-Roe vs. Wade era? How does having a high-risk prenatal diagnosis due to SCD further impact Black women’s choices after receiving NIPT results? How might anti-Black racism in scientific research and healthcare, explicit or implicit, influence Black women’s NIPT decision-making?* Notably, it is unclear whether advances in prenatal screening results for SCD will be beneficial and/or inflict harm on a medically vulnerable population facing serious social constraints.

### Bioethical considerations as NIPT advances to screen for SCD using cell-free DNA

Given the significant and well-documented health inequities affecting Black women’s reproductive health as well as the overlap for people living with and affected by SCD, our study underscores the need for additional bioethical considerations. Relatedly, what needs to be acknowledged in bioethical discourse is the lack of inter-disciplinary agreement on the impact of NIPT on Black women with genetic conditions. For instance, there is consensus across scientific fields regarding the existence of maternal and child health disparities, but a lack of consensus on how advances in NIPT screening will mitigate or exacerbate health disparities among Black women. This lack of synergy, perhaps as advances in NIPT increase, further affects the psychological and social experiences of Black women’s reproductive healthcare more broadly. Our results further highlight the need for a deeper NIPT discourse and analysis related to the moral experiences, dilemmas, and realities of minoritized individuals and communities plagued by structural injustice (Fletcher, Ray, et al. 2022). Based on our results, we gained additional insight from patients to ethically frame language that better aligns with the lived experiences of SCD, specifically in regards to framing SCD as a genetic abnormality. For example, if SCD patients do not use such language to describe SCD as an abnormality in their everyday lived experiences, then this has broader implications for framing NIPT SCD in clinical and scientific industries.

To advance NIPT screening equity among Black women, we recommend the following: First, there is a need for an expanded reproductive ethics framework that amplifies how NIPT advances might restrict informed and autonomous reproductive decision-making for Black women with chronic conditions, including Black women with SCD (Fletcher et al. 2021). Second, we need to critically acknowledge the lived experiences of Black women with and without SCD, particularly the complex intersections of anti-Black racism, social-classism, gender-based violence, medical oppression and debilitating health statuses in the context of NIPT advances (Fletcher, Thomas, et al. 2022; Thomas 2022). Third, we must consider that empirical research on NIPT predominantly enrolls White women (Chetty, Garabedian, and Norton 2013; Fletcher, Thomas, et al. 2022; Thomas et al. 2023). In other words, we need more studies that include, if not oversample, Black women to understand the possible effects of NIPT uptake on ongoing reproductive health disparities while also examining what contributes to minoritized patients’ lack of confidence in the healthcare system (Sederstrom and Lasege 2022). Genomics research, furthermore, fundamentally ought to institutionally prioritize reproductive bioethics related to understanding the varied lived experiences of Black women with and without genetic conditions. Thus, this work has important implications for expanding reproductive ethics discourse and praxis to center Black women in normative and empirical scholarship by conceptually aligning with justice-oriented frameworks, such as intersectionality, anti-Black racism, and constrained choice theory in the context of NIPT (Bird and Rieker 2008; Collins 2008; Crenshaw 2017).

### Strengths and limitations

One of the major strengths of our study is the large number of interviews conducted (*N* = 40), which captured a wide variety of perspectives highlighted by Black women in the US. This is a strength because it also attempts to mitigate the portrayal of Black women as a monolithic group in empirical NIPT studies. Additionally, this contributing factor distinguishes our research process by ultimately aiming to promote anti-Black racism by centering Black women’s reproductive health narratives in scientific data (Adams and Thomas 2018; Thomas et al. 2021). Indeed, because categorizing Black women into homogeneous social groups often fails to capture their intersectional identities and diverse-lived realities.

Even during the COVID-19 pandemic, recruitment efforts and response rates were relatively high. Although this was not a clinical study, we observed that Black women desired to participate, helping researchers understand the intersection between SCD and reproductive healthcare. This is contrary to the notion that Black patients are reluctant to participate in scientific studies because of mistrust (Thomas 2022; Wilson 2022). Many Black women research participants desire to share their experiences to inform the development of new reproductive technologies and ultimately reduce the burden of health inequities in their communities.

The limitations include the fact that we focused solely on SCD vs. non-SCD group comparisons and did not include other clinical and social identity groups in this analysis (e.g., sickle cell trait). Ongoing analysis of our dataset will inform future research directions and translational approaches.

## Conclusion

Now more than ever, examining the lived experiences of race, racism, gender-based violence, medical oppression, socioeconomic class discrimination, and racialized conditions such as SCD is critical when examining NIPT decision-making. Assessing Black women’s perceptions of NIPT utility can further help identify potential areas for genetic counseling, intervention strategies, and improvements in reproductive healthcare for historically minoritized groups. This requires a call to action on the part of researchers to ethically engage more Black women in research to dehomogenize the narrative of a single lived experience.

As NIPT advances to screen for SCD, reproductive equity must also advance. Ultimately, we hope that our study underscores the need to prioritize empirical studies that investigate Black women’s perceptions before, during, and after clinical encounters, particularly as NIPT screening for fetal SCD reaches the clinical market. Investigating the nuances within the perceptions of patients regarding NIPT and NIPT’s potential for advancing fetal or SCD screening can offer critical opportunities to alleviate the severity of constrained choices and expand reproductive equity. Bioethicists and Ethical, Legal, and Social Implications (ELSI) scholars are uniquely positioned to lead research at the intersection of new genomic technologies and maternal and child health to advance reproductive health justice.

## Supplementary Material

Supplementary Appendix A

Supplementary Appendix B

## Figures and Tables

**Table 1.** Characteristics of study participants.

Study Participant, Characteristics	SCD (*n* = 19)	No SCD (*n* = 21)
Average Age	35.79	31.29
Maternal Status		
Yes	7	10
# of Children	8	22
No	11	11
Pregnancy Loss	1	–
Genotype		
HbSS	13	N/A
HbSC	5	N/A
HbSβ+-thalassemia	1	N/A
Race [self-reported]		
AA and/or B	17	21
Afro-Latinx	1	–
Afro-Caribbean (Haiti, Trinidad, Tobago, Panama)	–	–
African (Ghanian, Nigerian)	1	–
Family History/Place of Origin [self-reported]		
US-Based/US-Born	10	12
Caribbean	4	3
Central America	1	–
Africa	3	1
Multiple	1	2
Unreported	–	3
Gender (Women), Sexual Orientation		
Heterosexual	19	16
Pansexual	0	1
Lesbian /or Bisexual	0	1
Unreported	–	3
Insurance Status (Proxy for SES)		
Private/Employer-provided	11	14
Public/Medicaid	8	6
No Coverage	–	1
Education Levels (Highest Level Indicated)		
High School Diploma or Equivalent	2	2
Some College	6	6
Associate’s or Bachelor’s Degree	5	5
Master’s Degree	5	6
Professional Degree	1	1
Doctoral Degree	–	1
