# Supplementary material for: Patient Perceptions on the Advancement of Noninvasive Prenatal Testing for Sickle Cell Disease among Black Women in the United States
Source: AJOB Empir Bioeth. Author manuscript; Available in PMC 2025 May 7. (PMC12057698; doi:10.1080/23294515.2024.2302996)
Supplement: Supplementary Appendix A [file NIHMS1966157-supplement-Supplementary_Appendix_A.pdf]

APPENDIX A

Version date: May 11, 2021

INTERVIEW PROTOCOL QUESTIONS

<u>Theme #1: Knowledge of NIPT</u>

<u>Broad Question (Pre-NIPT Material):</u>

1. **What does prenatal care mean to you?**
2. **When you hear "invasive prenatal testing," what does it mean to you?**
3. **When you hear "non-invasive prenatal testing," what does it mean to you?**
    - *<u>Probe-a</u>: Tell me what you know about Non-invasive prenatal testing (NIPT)? (before prompt)*
    - *<u>Probe-b</u>: Can you give me an example of how you came to know this information? From whom? when?*
    - *<u>Probe-c:</u> Based on what you already know (or do not know), what do you think about NIPT?*

<u>Broad Question (Post-NIPT Material):</u>

4. **Take about 5-10 minutes (or as much time as needed) to read through this brief NIPT material. Have you seen these any of these NIPT materials before? [Y/N]**
    - *<u>Probe-a</u>: After reviewing the NIPT material, tell me your thoughts about NIPT?*
    - *<u>Probe-b</u>: After reviewing the NIPT material, what do you like about NIPT? What do you dislike?*
    - *<u>Probe-c</u>: After reviewing the NIPT material, what are your thoughts about having NIPT in prenatal care?*

<u>Theme #2: Sickle Cell Disease / Illness Experience</u>

Clinical Vignette / Example Patient-Story

Tiffany is 7 weeks pregnant when her provider discusses prenatal genetic testing. Tiffany is aware that she is a carrier of sickle cell trait (SCT) as she was identified through her own screening as a newborn. Tiffany's NIPT results were *positive for her newborn having sickle cell disease*. The genetic counselor informs her that she would have to take further diagnostic testing (e.g., amniocentesis or CVS) to confirm the results. However, further testing would have to be invasive, which can increase the likelihood of a procedure-related miscarriage.

- *<u>Probe-a</u>: What do you think Tiffany should do? What would you do?*
- *<u>Probe-b</u>: Would you use NIPT to see if sickle cell disease could be detected in the pregnancy? Why yes? Why not?*
- *<u>Probe</u>-c: What would you do after learning about your results?*

Specific Questions for: Sickle cell population [n=20]:

1. *How do you feel about reproductive healthcare in the United States?*
2. *What is do you consider the most, when thinking of having a child?*
3. *How would NIPT be helpful to SCD pregnancies?*
4. *How would NIPT be harmful to SCD pregnancies?*
5. *What should prenatal care be like for you?*

Theme #3: Informed Consent

5. **Broad Question: What has been your experience with informed consent for procedures such as blood test and screening procedures (e.g., amniocentesis or CVS)?**
    - *Probe-a: How should you be informed about NIPT prior to testing? From whom? When?*
    - *Probe-b: How do you perceive saying "no" to NIPT? What does say "no" mean to you during prenatal care?*
    - *Probe-c: How do you perceive saying "yes" to NIPT? What does saying "yes" mean to you during prenatal care?*

Theme #4: Racialization / Additional Social Identities at Clinical Encounters

6. **Broader Question: As a Black woman, do you think there are unique issues you face regarding prenatal care?**
    - *Probe-a: How do you feel about NIPT as a Black woman?*
    - *Probe-b: What could be helpful or helpful about NIPT for Black women in the United States?*
    - *Probe-c: Do you think race plays a role in NIPT?*

7. **Broader Question: Have you ever experienced discrimination in your healthcare? [Y/ N = depending on probe]**
    - *Probe-a: Can you tell me about those experiences? Can you walk me through that moment?*
    - *Probe-b: Have you ever experienced racism during a health appointment or prenatal care appointment?*
    - *Probe-c: Can you give me an example?* Can you walk me through that moment?
    - *Probe d: Which types of health appointments would you say that you experience the most discrimination?*

Theme #5: Receiving Results

8. **Broader Question: Have you ever received any results from a reproductive health appointment (e.g. annual exams, PAP smears, breast exams)? How did it make you feel?**
    - *Probe-a: What would you if your results came back positive? Or negative?*
    - *Probe-b: How would you make this decision?*
    - *Probe-c: What makes this decision easier or challenging for you?*
    - *Probe-d: Who (in your care-network) would help you make this decision?*

9. Last Question: Is there anything else you would to add?

[End of Interview Questions]
