# Supplementary material for: Patient Perceptions on the Advancement of Noninvasive Prenatal Testing for Sickle Cell Disease among Black Women in the United States
Source: AJOB Empir Bioeth. Author manuscript; Available in PMC 2025 May 7. (PMC12057698; doi:10.1080/23294515.2024.2302996)
Supplement: Supplementary Appendix B [file NIHMS1966157-supplement-Supplementary_Appendix_B.pdf]

The American College of Obstetricians and Gynecologists
WOMEN'S HEALTH CARE PHYSICIANS

Cell-Free DNA Prenatal Screening Test

How the Test Is Done

What is it?

The cell-free DNA prenatal screening test screens for certain conditions caused by an abnormal number of chromosomes. It does not test for all types of chromosomal disorders.

When can it be done?

A cell-free DNA test can be done as early as 10 weeks of pregnancy and up until delivery.

How is it done?

Some of the genetic material (DNA) from the pregnancy circulates in the pregnant woman's blood. The cell-free DNA test is done on a sample of her blood.

Screening tests are used to estimate whether your fetus is at higher risk or lower risk of having a certain condition.

Diagnostic tests can give a definite answer about whether the fetus has a certain condition. These tests include amniocentesis or chorionic villus sampling (CVS).

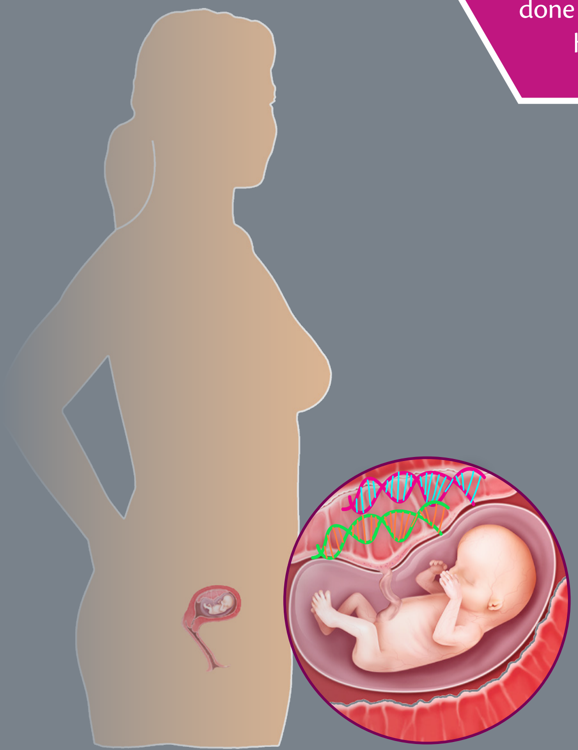

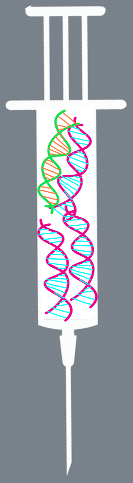

A blood sample is taken from the pregnant woman that contains her DNA and DNA from the pregnancy. →

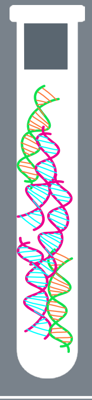

The sample is analyzed in a laboratory to check for an abnormal amount of DNA from chromosomes 21, 18, and 13.

Major conditions screened for:

- Trisomy* 21 (Down syndrome)
- Trisomy 18
- Trisomy 13

Conditions not screened for:

- Problems that are screened for by ultrasound, such as neural tube defects, heart defects, and abdominal wall defects
- Many other chromosomal and genetic disorders

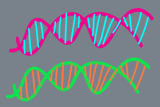

= woman's DNA
= DNA from the pregnancy

*Trisomy means that there are three copies of a particular chromosome instead of the normal two copies. For instance, trisomy 21 means that there are three copies of chromosome 21.

PFSI008: This information was designed as an educational aid to patients and sets forth current information and opinions related to women's health. It is not intended as a statement of the standard of care, nor does it comprise all proper treatments or methods of care. It is not a substitute for a treating clinician's independent professional judgment. Please check for updates at www.acog.org to ensure accuracy.

Copyright May 2019 by the American College of Obstetricians and Gynecologists. All rights reserved. No part of this publication may be reproduced, stored in a retrieval system, posted on the internet, or transmitted, in any form or by any means, electronic, mechanical, photocopying, recording, or otherwise, without prior written permission from the publisher.

National Society of Genetic Counselors

Society for Maternal·Fetal Medicine

The American College of Obstetricians and Gynecologists
WOMEN'S HEALTH CARE PHYSICIANS

Cell-Free DNA Prenatal Screening Test

Understanding Your Results

What do the results mean?

If you get a positive result, how likely is it that the fetus has the disorder? This is called the positive predictive value (PPV) of the test.

If you get a negative result, how likely is it that the fetus does not have the disorder? This is called the negative predictive value (NPV) of the test.

For the cell-free DNA test, the positive predictive value depends on how frequently the disorder occurs in a group of people similar to you.

For Example: Trisomy 21 (Down Syndrome)

High-risk* group

The disorder occurs more frequently in this group.

Positive result

PPV = 83%

Out of 100 women with a positive result, 83 will have babies with Down syndrome, and 17 will not have babies with Down syndrome.

Negative result

NPV = 99+%

Women with a negative result will only rarely have a baby with Down syndrome.

Low-risk group

The disorder occurs less frequently in this group.

Positive result

PPV = 33%

Out of 100 women with a positive result, 33 will have babies with Down syndrome, and 67 will not have babies with Down syndrome.

Negative result

NPV = 99+%

Women with a negative result will only rarely have a baby with Down syndrome.

Sometimes the test does not yield a result or is indeterminate. In this case, you should receive further genetic counseling.

An ultrasound exam and diagnostic testing also should be offered due to an increased risk of a chromosomal disorder.

*You are at "high risk" if you are 35 years or older; you have had an ultrasound exam that shows a possible problem with the fetus; you have had a previous child with one of these disorders; you have a chromosomal problem that increases your risk of having a child with trisomy 21 or trisomy 13; or you have had a positive first-trimester or second-trimester screening test result.

BOTTOM LINE:

- Cell-free DNA testing is a very good screening test to detect common chromosomal disorders, but it has limitations.
- A negative result does not rule out the possibility of having a baby with a chromosomal disorder or other disorders that the cell-free DNA test does not test for.
- If you have a positive result, a diagnostic test is needed to determine if the fetus is truly affected.
- If you have cell-free DNA screening, a blood test or ultrasound exam should be offered to screen for neural tube defects and other conditions.

PFSI008: This information was designed as an educational aid to patients and sets forth current information and opinions related to women's health. It is not intended as a statement of the standard of care, nor does it comprise all proper treatments or methods of care. It is not a substitute for a treating clinician's independent professional judgment. Please check for updates at www.acog.org to ensure accuracy.

Copyright May 2019 by the American College of Obstetricians and Gynecologists. All rights reserved. No part of this publication may be reproduced, stored in a retrieval system, posted on the internet, or transmitted, in any form or by any means, electronic, mechanical, photocopying, recording, or otherwise, without prior written permission from the publisher.

National Society of Genetic Counselors

Society for Maternal-Fetal Medicine
